# Neuroprotective and consequent neurorehabilitative clinical outcomes, in patients treated with the pleiotropic drug cerebrolysin

**Published:** 2009-11-25

**Authors:** G Onose, DF Mureşanu, AV Ciurea, CD Chendreanu, AS Mihaescu, DC Mardare, I Andone, A SpȦnu, C Popescu, A Dumitrescu, M Popescu, V Grigorean, B Ungur, F Marinescu, I Colibaşeanu, L Onose, M Haras, A Sandu, T Spircu

**Affiliations:** * ‘Bagdasar–Arseni’ Teaching Emergency Hospital, BucharestRomania; ** ‘Carol Davila’ (State) University of Medicine and Pharmacy, BucharestRomania; *** The Neurological Clinic of ‘Iuliu Hatieganu’ (State) University of Medicine and Pharmacy, Cluj–NapocaRomania; **** The 1^st ^ Clinic Division of Neurosurgery, ‘Bagdasar–Arseni’ Teaching Emergency Hospital, BucharestRomania; ***** The Neurosurgery Division of the County Hospital, PitestiRomania; ****** The Medical Service of Metrorex, BucharestRomania

## Abstract

**Background**: Discovery of neurotrophic factors–emblematic: the nerve growth factor (NGF)–resulted in better approaching central nervous system (CNS) lesions. Recently, another crucial property has been unveiled: their rather unique pleiotropic effect. Cerebrolysin is a peptide mixture that penetrates the blood–brain barrier in significant amounts and mimics the effects of NGF.

**Methods**: Comparative analysis: Cerebrolysin treated (10 ml x 2/ day, i.v. x 3 weeks) vs. non–treated, in patients (all received aside, a rather equivalent complementary, pharmacological and physical, therapy). Two lots of patients, admitted in our Physical and Rehabilitation (neural–muscular) Medical–PR(n–m)M–Clinic Division, during 2007–2009: 69 treated with Cerebrolysin (22 F, 47 M; Average: 59.333; Mean of age: 61.0 Years old; Standard deviation 16.583) and 70 controls (41 F, 29 M; A: 70.014; M.o.a.: 70.5 Y.o.; S.d.: 6.270) were studied. The total number of assessed items was 13: most contributive in relation with the score of Functional Independence Measure at discharge (d FIM), were: admission (a FIM), number of physical therapy days (PT), number of hospitalization days (H), age (A) and–relatively–days until the first knee functional extension (KE). Concomitantly, the main/ key, focused on neuro–motor rehabilitative outcomes, functional/analytical parameters, have been assessed regarding the speed in achieving their functional recovery.

**Results**: Concerning d FIM, there have not been objectified significant differences between the two lots (p=0.2453) but regarding key, focused on neuro–motor rehabilitative outcomes, functional/analytical parameters: KE (p=0.0007) and days until the first time recovery of the ability to walk between parallel bars (WPB–p=0.0000)–highly significant differences in favor of Cerebrolysin lot resulted.

**Conclusion**: Cerebrolysin administration, as neurorehabilitative outcomes, proved to hasten, statistically significant, especially the recovery of some critical, for standing and walking, parameters. Thus encouraged, we have now initiated a comprehensive national, 5 year retrospective, multi–centre – based on unitary data acquisition frame and mathematical apparatus–study, to evaluate the results of the treatment with Cerebrolysin in traumatic brain injuries (TBI).

## Introduction

In 1986, Rita Levi–Moncalcini (from The Cellular Biology Institute, Rome, Italy) and Stanley Cohen (from the Vanderbilt University School of Medicine, Nashville, USA) received the Nobel Prize for discovering neurotrophic factors: the nerve growth factor–NGF– respectively, the epidermal growth factor–EGF.

Since then, many other neurotrophic factors have been identified: they are polypeptides, naturally synthesized by all types of cells within the CNS and also by other tissues. Their activity is essential for the NS development (they stimulate cell proliferation and differentiation, respectively axonal and dendritic growth), for the neural cells' natural survival in the absence of injury/resistance to noxious factors and for their phenotype retaining, during lifetime. 

Neurotrophic factors stimulate neural plasticity and synaptic activity, and therefore are important for both: learning processes and for the NS's impressive ability to spontaneously reorganize and thus, clinical adapt/(limited) self–recover, after different injuries.

The discovery of neurotrophic factors–emblematic: NGF–resulted in better approaching CNS lesions. Recently, another crucial property has been unveiled: their rather unique pleiotropic effect [1] – i.e. a combined, complex neuroprotection and neurotrophicity (including neural plasticity) stimulation.

CNS injuries are divided into two main categories: primary–which occur (mainly) at the moment of a trauma–and secondary ones, that develop after the initial injury, as a consequence of a complex and rather specific to CNS, patho–physiological events' cascade; they produce effects that may continue for a long time. The secondary injury process (synthetically including: excessive synthesis of nitric oxide and oxidative stress, microglia activation, local inflammation, disturbance of microcirculation, blood–brain barrier dysfunction and the most recently acknowledged ‘delayed mechanisms of cell death’ [2,3,4, 5] ) leads in vicious circles, to disastrous consequences:

- neuronal necrosis;
- neuronal apoptosis;
- scar and/or cyst/ hygroma formation–with further– pathogenic effects on CNS tissue;
- demyelination;
- disruption of morpho–functional nerve pathways (disconnection) and/or functional uncoupling, such as diaschisis.

Thus, minimizing the secondary damage ‘cascade’ could result in maximizing post–injury favorable evolution/recovery, including more rapid and consistent neuro–rehabilitative outcomes. 

Therefore, the CNS intimate mechanisms of the secondary injuries are, at present, main targets for modern, including pleiotropic, complex therapies. CNS main pathways for the secondary damage (occurring in the affected area and in its neighborhood):

1. Breakdown of the primary traumatized area's cells
2. Breakdown of the myelin sheath's structure.
3. Release, from inside the disrupted CNS cells–mitochondria are important sources–of reactive oxygen species (ROS).
4. Microglia activation including pro–inflammatory, with subsequent delivery of cytokines from these injured structures and of wall components, as well (together– in vicious circle) with supplementary amounts of ROS – as a result of ROS peroxidation lesions of membranes' phospholipids (under excessive, post tissue injury–including neural–metabolic, mitochondrial/ cell hyperactivity).
5. Oxidative stress–the (hyper) local metabolic generation of ROS and physiological antioxidants' depletion, with subsequent alteration of some gene expression functions (especially for factors/ transcriptional mechanisms type: NF–kB, PPAR, AP–1) and thus priming, including synthesis sequences, that stimulates production of pro–inflammatory cytokines–especially interleukins IL–1, IL–6 and tumor necrosis factor (TNF) alpha–respectively,  with concomitantly reduction of related molecules synthesis (but with anti–mediator role–for example: IL–2).
6. Immune imbalance/inflammation
7. Disorder of local microcirculation and integrity of the blood–brain barrier (with consecutive regional ischemia and edema)
8. Electrolyte disorders, including massive edema–CNS tissue swelling–induced by suddenly installed osmolysis (often one of the direct effects of primary injury, too) subsequent to the affected cells which die passively – thus, violent osmolysis is also, in such circumstances, a main necrosis mechanism: in necrosis it is the cellular edema which leads to osmolysis, with the cell passively dying off
9. Increase of the nervous tissue metabolism, including oxygen consumption, thus resulting, in the vicious circle, of its sensitivity to hypoxia and–once more–ischemia
10. Large amounts of tiny molecules – Transient Receptor Potential Member (TRPM) 7–invade the normal surrounding neurons' membrane surfaces and very probably, mainly through adenil–cyclase, dramatically enhance their oxidative metabolic activity, resulting in more ROS that propagates the damages to an extensive cell (both) apoptosis and necrosis process, in the unaffected neighborhood, too
11. Resulting in a relative excess of exciting neurotransmitters and massive influx of intracellular (toxic/metabolic destructive) calcium ions (see further).
12. Sequential activation of key–role genes, including (most dangerous for a non–regenerating tissue, like CNS–as neurons lack centrosomes) those for  apoptosis–triggering the ‘mechanisms of delayed cell death’: programmed cell suicide and apoptosis–like processes–most recently emphasized, having a longer display and being produced at an intimate level, mainly through metabolic disturbance of aggregated proteins involved in the deep mechanisms of cellular reproductive cycles/ vitality–survival [1,6,7].
  Briefly, it is worth to synthetically emphasize some of the main beneficial actions but it also limits/side effects–related to the pleiotropic effect subject matter– of one of the most studied and controversially used–including in CNS acute lesions–drug, with strong anti–inflammatory properties: metilprednisolone (MP). The most important action of MP in the acute stage of injury is to inhibit the lipid peroxidation induced by ROS, thus limiting the secondary damage. The antioxidant effects of MP are not mediated via glucocorticoid receptors: other steroidal anti– inflammatory drugs (SAIDs) do not possess similar antioxidant activity [8].
  MP interferes with other neural pathological pathways, too:
  - decreases the arachidonic acid release;
  - lowers the cellular inflow of calcium ions and subsequently, the apoptosis processes;
  - decreases anaerobic metabolism and prevents (toxic) lactate/acidosis accumulation;
  - minimizes neurofilaments degeneration;
  - reduces post–traumatic nevrax  edema and its compressive consequences;
  - helps maintaining the neuronal membrane potential and the synaptic transmission
  Adverse actions: in the recent years, the use of high–dose MP has become controversial, mostly based on the risk of serious side effects versus a modest neurological benefit. The steroid side effects are prominent when the treatment is extended beyond 24 hours: pneumonias, septic shocks, wound infection, delayed wound healing, pressure sores, hyperglycemia, deep–vein thrombosis, gastro–intestinal bleedings [9– 12].
  Other limitations to high–dose MP therapy: the neuroprotective properties of MP have a sharp U–shaped dose–response curve, that requires careful dose calculation; initiation of treatment beyond the 8–hour opportunity window can exacerbate damages: inhibition of axonal budding and synaptogenesis. Considering the long term neurological outcome, the potential of the steroid to attenuate post–injury neural plasticity is probably the most serious concern regarding the administration of high doses of MP.
13. Higher concentrations of calcium ions, extruded on the exterior of the nerve cells' break, flood the interior of these (and also other, non– affected) neurons [13]. In the attempt of regaining the pressure's balance of the ionic concentrations, calcium sets off a series of self–destructive cellular events, among which very important is: its interference, at mitochondrial level, with the electron transport/acceptor chain, thus resulting in a greater amount of ROS (Fig 1).

**Fig 1:**
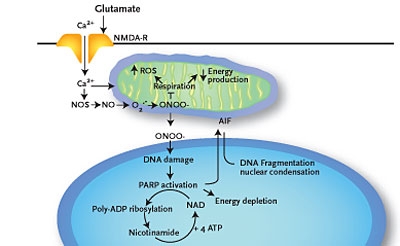
Glutamate increases Ca^2+^ cellular influx, activating neuronal nitric oxide–synthetase (nNOS); this enzyme, in the presence of Ca^2+^ absorbed at mitochondria level, converts nitric oxide (NO) in peroxinitrite (ONOO^– ^): one of the most toxic ORS; Ca^2+^ and ONOO^–^, apparently paradoxical, blocks the mitochondrial breathing (in terms of oxidative metabolic hyperactivity, which is induced by Ca^2+^); concomitantly/ consequently, at the respiratory chain level, increases the production of ROS. It results: energetical mitochondrial collapse, damaging (predominantly by peroxidation) the membranar lipids – with propensive permeabilisation getting out from mitochondrias and translocating to the nucleus of AIF – and DNA by ROS (mainly ONOO^–^) and respectively, secondary hiperactivation of poly–ADP–ribozo– polymerase (PARP) –1 enzyme; the latter convey the signals of cell suicide by engramated, preformated way on nuclear level, through chemical–energetical revolving plate's depletion, represented by nicotine–amide– dinucleotide (NAD)+/ATP, resulting  a proapoptotic effect, synergically in such cases, with the one of  Endonuclease G (Endo G) – after Hong cited by Blackman S A, 2005 [1,3]
14. Injury releases amounts of different neuro–transmitters higher than usual: catecholamines, endorphins, serotonin and (most ‘dangerous’, especially in the early post–injury stages) glutamate, the main excitatory normal neuro–transmitter; in large, abnormal, amounts without enough valid neurons to respond to, glutamate  expresses  its  toxicity  by overloading intact remaining neuronal  circuits.
15. Calcium, ROS and endogenous tissue enzymes (proteases, phospholipases, lipooxygenases, cyclooxigenases) work in concert to destroy dead or dying cells.
16. Prostaglandins produce chemotactism and (supplementary) local/regional vasoconstriction/ ischemia.
17. The oxygen breakdown of essential cell lipids (lipid peroxidation) and the other previously exposed pathways, lead, into a vicious circle, to more swelling, by water entering CNS–especially the brain–tissue, from the blood and cerebro–spinal fluids, thus leading, to more cell breakdown and more secondary release of toxic substances, that again, affect blood flow.
18. By–products of many of these reactions, of the events' cascade pathways, also stimulate the glial cells (first of all the astrocytes, which have a complex, vital role–‘housekeeping’–within the NS): to emit ‘signaling’ molecules, instructing to proliferate, in the attempt to replace/repair the destroyed/lost nevraxial tissue; this results mainly, in gliosis and (unfortunately) in scars (a major source for further limits in CNS recovery).

Additionally to the lack of self repair significant skills and to the extensive secondary damages pathways, in the CNS, for reasons yet unclear, there are also strong inhibitors mainly of axonal re–growth. Hence, among the main nevraxial–afore emphasized–limits in self recovery after injuries, there are also some inner obstacles that prevent CNS cells' regeneration, generically called the ‘braking’ machinery in neurons: tightly related to the Neurite Outgrowth Inhibiting (NOGO) protein and receptors (Schwab et al., since the middle eighties [14,15, 16]) and more generally, to the rho family of receptors(17); this family of receptors  relays on a protein called TAJ or TROY and another one–p75–that acts as an important part of the same family of receptor complex proteins–called TNF receptors–on neurons, responding to growth–inhibitory molecules in myelin and thus, preventing the cable–like axons' (re)–growth of injured neurons in CNS: acceptance of these inhibitory molecules, like a key fitting a lock and switched–on, results in inhibitory signaling, within the neuron [18].

Today, more than 500 substances are or have been studied for neuroprotective properties.

As traumatic and ischemic injuries in both, the brain and the spinal cord, entail/contain very resembling/rather overlapping–as mechanisms types within the patho–physiological events' cascade, leading to secondary lesions–the ‘good news’ is: neurotrophic and especially, pleiotropic substances, conceptually (and practically–growing evidences^19^) thus justify a quite large clinical utility spectrum. 

Cerebrolysin is a peptide mixture obtained by standardized enzymatic (proteo)lysis breakdown of purified porcine brain proteins. It consists of approximately 25% biologically active low molecular weight peptides and amino acids that are able to penetrate the blood–brain barrier in significant amounts and mimic the effects of NGF. 1 ml of injectable solution contains 215,2 mg of protein lysate and excipients (sodium hydroxide, water). The injectable solution does not contain proteins, lipids, or other antigenic molecules. 

As it will be seen further, it targets and counteracts many essential pathways of the secondary damage cascade and concomitantly, stimulates/ facilitates mechanisms of re–adapting and (limited) self repair in CNS injuries, i.e. the corollary –relatively rare and most beneficial–pleiotropic effect:

**Fig 2:**
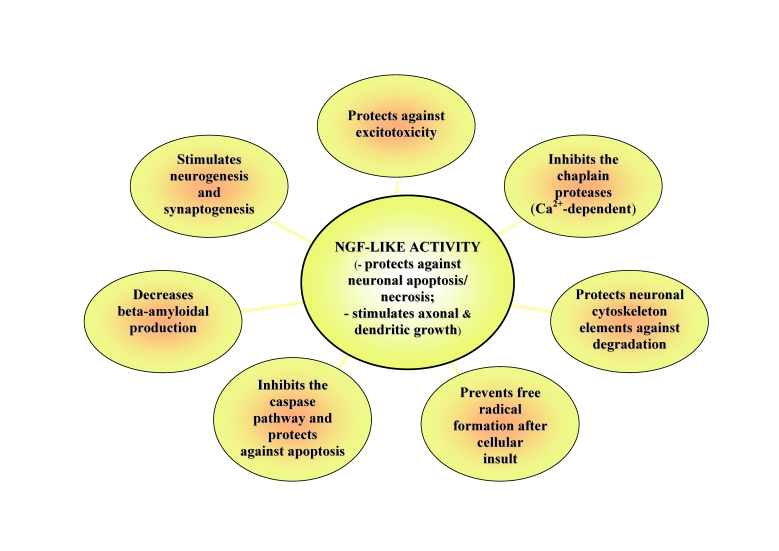
Correspondence between Cerebrolysin's main actions and pathways of the secondary injuries cascade it targets/ counteracts

**Fig 3:**
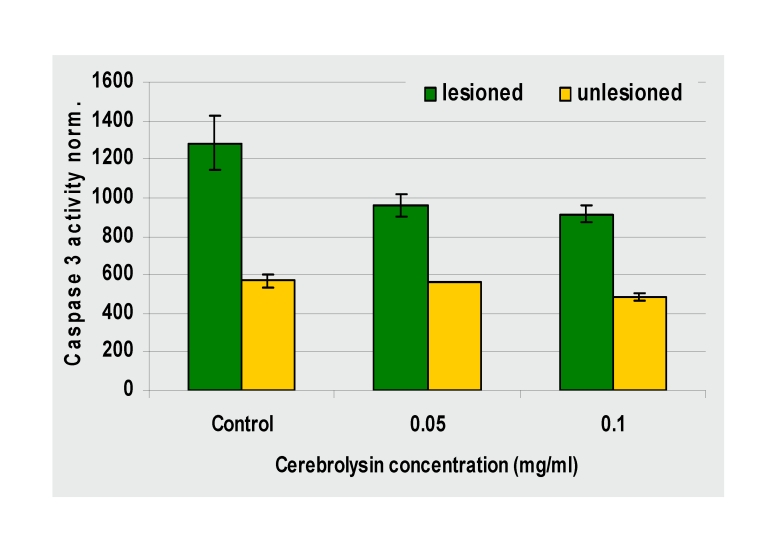
Capase inhibition by Cerebrolysin–Caspase 3 activity measured 48 hours after a lesion with 6µM ionomycin: Cerebrolysin's inhibition is dose–dependent (by courtesy of Ebewe)

**Fig 4:**
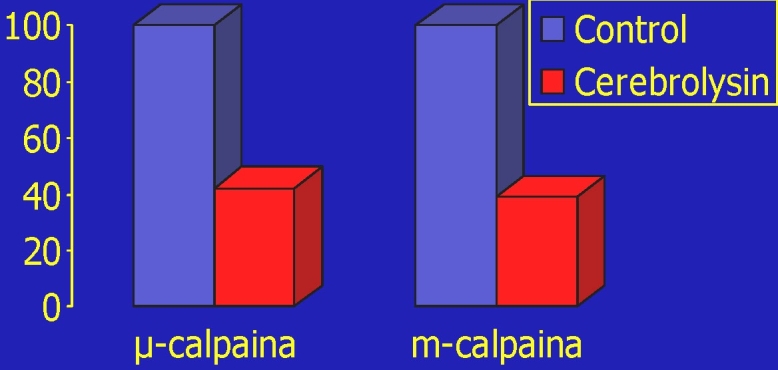
Calpain inhibition by Cerebrolysin [20]

- Cerebrolysin inhibits both µ–and m–calpain in an (also) dose–dependent manner. The protease inhibition is non–competitive and reversible;
- Therefore, Cerebrolysin protects the cytoskeleton elements susceptible to calpain degradation: in neuronal cell cultures, it reduces the loss of (morpho– functional very important) Microtubule–associated protein (MAP) 2, after a cell injury.

**Fig 5:**
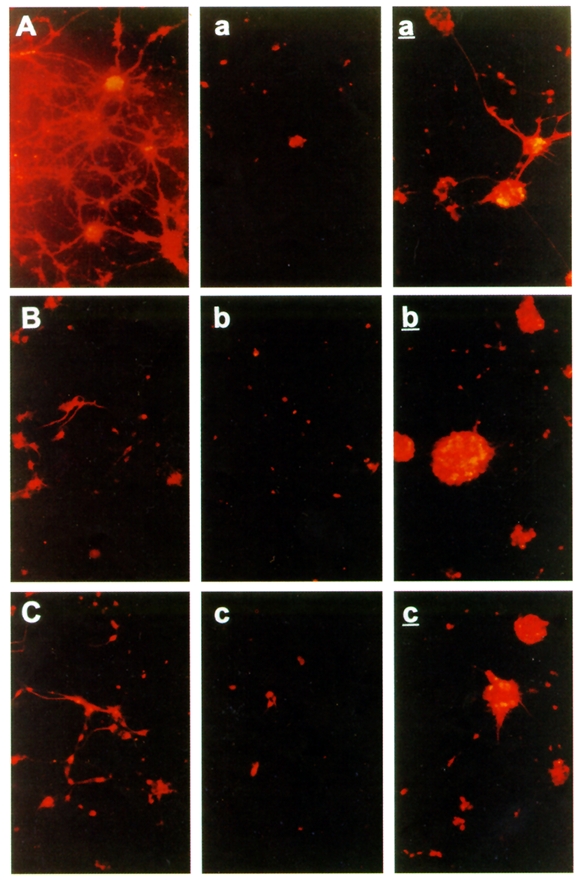
Neural cell cultures exposed to hypoxic lesions [21]–protection by Cerebrolysin of  the Microtubule–Associated Protein (MAP) 2–in vitro: A, B, C–non–lesioned controls; a, b, c–lesioned controls; a', b', c'–lesioned, Cerebrolysin treated

Below–some effective, intimate histopathological outcomes of the treatment with Cerebrolysin. [Fig 5]

**Indications:**As already emphasized, Cerebrolysin exhibits complex neuroprotective and neurotrophic–pleiotropic–actions. These effects have been investigated and confirmed in various cell culture and animal models of neuro–degeneration and ischemic injuries, as well as in clinical trials. 

The window of opportunity in acute stroke is considered to be of 24 hours. Clinical trials have demonstrated significant motor and cognitive improvements in stroke and dementia patients, leading to consistent amendments to their quality of life (QOL).

**Interactions:** Cerebrolysin should not be mixed in perfusion with neutral amino acid solutions. The doses of anti–depressive medication and particularly, of monoamine oxidase inhibitors (MAOIs) should be lowered if used in conjunction with Cerebrolysin.

The **side effects** of Cerebrolysin are infrequent and usually mild and transient: agitation (aggressiveness, insomnia, rarely hallucinations), confusion, tremor, allergic reactions–very rare, in our expertise (fever, skin reactions, pruritus, local vascular reactions, headache, neck pain, limb pain, lower backache, dyspnea, chills, shock–like state), vertigo, headache, hypertension or hypotension, hyperventilation, hypertonia or hypotonia, fatigue, depression, apathy, flu–like symptoms, gastro–intestinal troubles (loss of appetite, dyspepsia, diarrhea, constipation, nausea, vomiting), rapid injection may cause heat sensation, sweatiness, dizziness, rarely palpitations or cardiac arrhythmias, injection site reactions (irritation, pruritus, burning sensation).

**Contraindications** are: hypersensitivity to the protein lysate or to the excipients; epilepsy, especially grand mal convulsions (Cerebrolysin treatment may increase the frequency of seizures); severe or acute kidney failure; there is no available information on the safety of Cerebrolysin during pregnancy and lactation in humans, though animal studies found no toxic effects; some studies have shown that Cerebrolysin can be safely used in patients with acute hemorrhagic stroke

## Objective

The objective of this study was to assess the outcomes obtained in our PR(n–m)M Clinic Division with Cerebrolysin, compared to patients who did not receive such neuroprotective/neurotophic (pleiotropic) therapy.

## Material and methods

The study included two lots of patients, admitted during 2007–2009: 69 treated with Cerebrolysin (22 F, 47 M; Average: 59.333; Mean of age: 61.0 Years old; Standard deviation 16.583) and respectively, 70 – controls (41 F, 29 M; A: 70.014; M.o.a.: 70.5 Y.o.; S.d.: 6.270).

## Study design

A comparative analysis between Cerebrolysin (10 ml x 2/ day, i.v. x 3 weeks), vs. patients non–treated with Cerebrolysin (all the inpatients received aside, a rather equivalent complex, pharmacological and physical therapy).

The Cerebrolysin treated lot has been constituted on a random base, ‘naturally’ represented by the periods, within the duration of our study, when our hospital's pharmacy could supply this drug.

**Fig 6:**
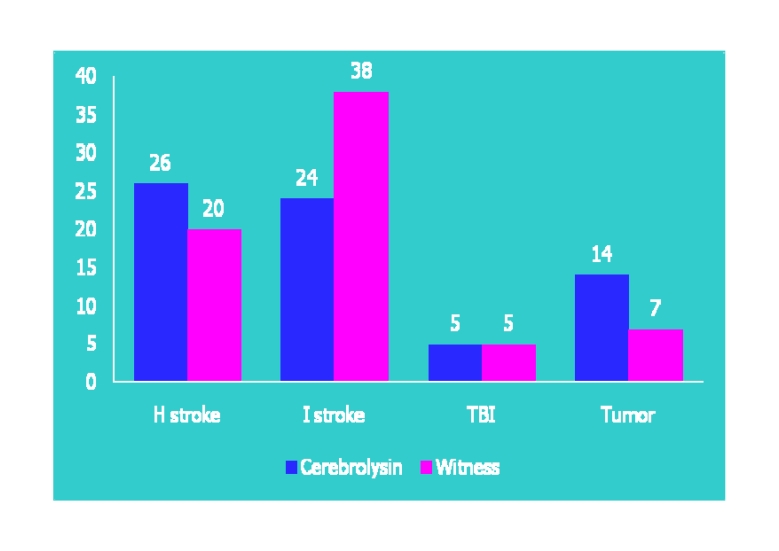
The distribution of the two studied lots, by etiology

All cases were admitted in PR (n–m)M Clinic Division, mainly in early subacute stage (between 10–14 days after the initial injury) transferred from the neurosurgery and neurology departments in Bucharest and the surrounding areas (70,65% cases), but also from the entire country–our Clinic is the National Reference Centre for Neurorehabilitation–or readmitted, within the first year after injury.

The total number of assessed items was 13, among which the most contributive, in relation to the score of the Functional Independence Measure at discharge (d FIM), according to our clinical rehabilitative results, were the first five of them:

- admission/ discharge Functional Independence Measure (aFIM)
- number of physical therapy days (PT/KT)
- number of hospitalization days (H)
- age (A)
- days until first knee functional extension (KE)
- days until the first walk between parallel bars (WPB)
- days until the first independent walk recovery (IWR)
- days until the first cane assisted walk recovery (CWR)
- days until the first stairs ascent /descend recovery (SR)
- etiology (E)
- gender (G)
- evolutive status at discharge (ES)

**Fig 7:**
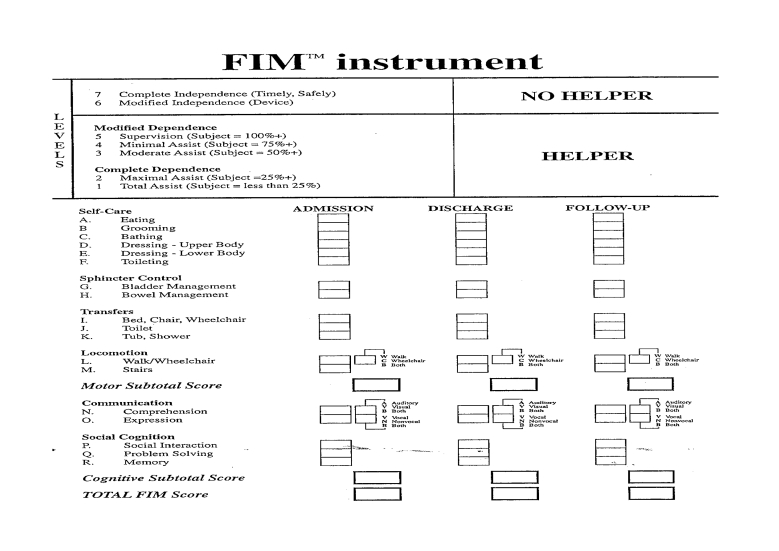
The (worldwide accepted as standardized assessment tool) Functional Independence Measure (FIM)

### Mathematical methodology

Our statistical analysis used, as desired, a differentiation method to assess the validity of outcomes, the T TEST; previously this entailed the mathematical evaluation of the two studied populations' normality of distribution. Hence, if a population is normally distributed–according to the frequency histogram and the following calculation tools: Min, Max, Aver., St. Dev.–it is to be expected strong validity results of the applied T TEST; if the population is not normally distributed– as it was, mostly the case of the assessed parameters within our study–we applied the CHI SQUARE TEST, by the frequency histogram–giving thus, potential for evaluation to more of our assessed parameters.

There have been done also correlation analysis, to objectify the statistical assessed variables/phenomena' dependence, between them, quantified by the calculated value–through the EPI INFO soft–of the correlation coefficients (positive or negative). Hence, once emphasized a certain dependence between variables, they had to be quantified by regression assays; as it is well known, there are simple regressions (with the generic formula: Y = a+bX, where: Y = the dependent variable; X = the independent variable; a and b = regression coefficients; b is also the straight line's slope, representing the amount in which Y's value changes when X's value varies by one unit) and multiple ones: applied to this study, all the independent variables (a FIM, PT/KT, H, A, KE, WPB, SR, EXR, CWR, IWR, G, E) are simultaneously intervening. Thus, we were interested in the construction of a mathematical model/ equation, in which the dependent variable Y is d FIM and the independent variables are: X_1_ (a FIM), X_2_ (PT/KT) , etc. The resulting formula was: Y = B_0_ + B_1_ X_1_ + B_2_ X_2_ + … + B_n_ X_n_ – multiple regression–where:Y = the dependent variable (d FIM), B_0_ = the tabular appropriate correlation/ regression coefficient, B_1_ = the correlation/ regression coefficient of a FIM, etc.

**Fig 8:**
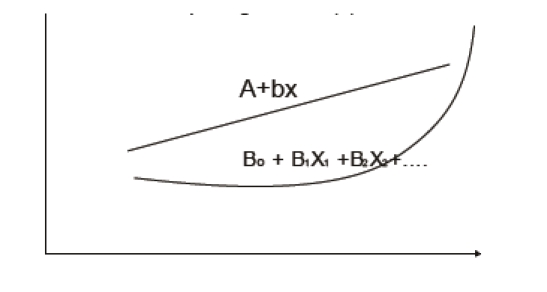
The curves for simple and respectively, for multiple regression

## Results and discussions

First, two studied populations' normality distribution level has been assessed, with respect to each main parameter, starting with the first–focused (but) non functional/ analytical–one, PT/KT:

**Fig 9:**
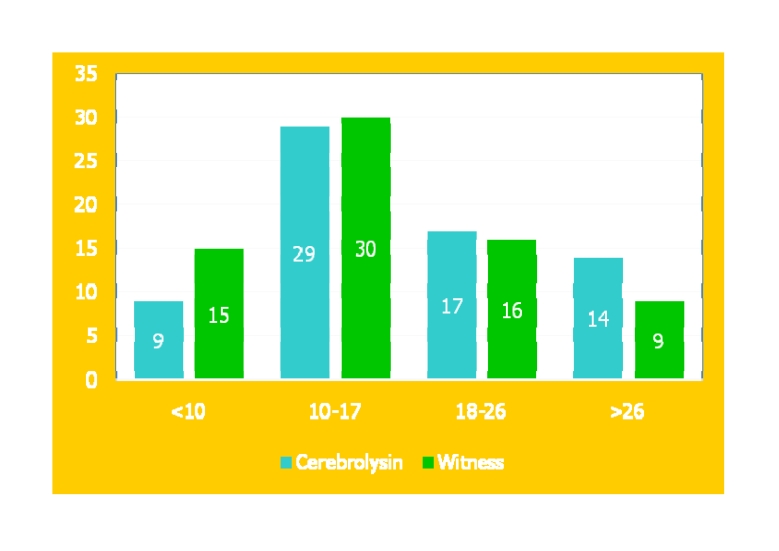
Frequency  distribution

**Table 1:** Normality distribution values of the two assessed populations, regarding PT/KT parameter

	**PT/KT** Cerebro
Average	18.92753623
St. dev.	10.96695416
	**PT/KT** witness
Average	16.6
St. dev.	**12.50437605**
	
**p** value	0.122600944

**Table 111:** 

	freq. **PT/KT** cer.	freq. **PT/KT** witn.			normal cer.	normal witn.
8,5	0,0580	0,2286	4	16	0,185184	0,206929
16,5	0,4638	0,3143	32	22	0,283971	0,255226
24,5	0,2754	0,2714	19	19	0,255771	0,209057
32,5	0,0435	0,1286	3	9	0,13531	0,113722
40,5	0,1159	0,0429	8	3	0,042045	0,041083
56,5	0,0145	0,0000	1	0	0,000823	0,00157
64,5	0,0145	0,0000	1	0	5,18E–05	0,000166
72,5	0,0000	0,0000	0	0	1,92E–06	1,17E–05
80,5	0,0000	0,0000	0	0	4,16E–08	5,45E–07
88,5	0,0000	0,0143	0	1	5,31E–10	1,69E–08
	1,0000	1,0000	69	70	0,910832	0,837621

As (also) emphasized by the histogram below, a statistically significant populations' normality distribution hasn't been observed:

**Fig 10:**
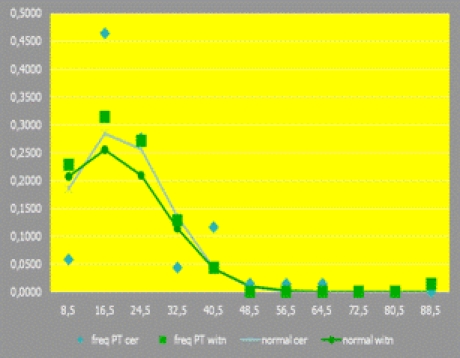
Histogram emphasizing the level of the two studied populations' normality distribution, regarding PT/KT

Accordingly, we proceeded to CHI^2^ TEST assay; the resulted p=0.152824, emphasizes that statistical significant differences between the two lots, regarding this parameter have not been objectified:

**Table 2:** The tabular form of the CHI^2^ TEST results regarding PT/KT

**PT/KT**	Cerebolysin	Witness
<10	9	15
10–17	29	30
18–26	17	16
>26	14	9
chi^2^ test val. **p**	0.152824	
0.5 val. **p**	0.076412	

Similarly, related to the frequency distribution, we comparatively analyzed the two populations' normality, regarding the – focused (but) non functional/ analytical–H parameter: 

**Table 3:** Normality distribution values of the two assessed populations, regarding H parameter

	**H**Cerebrolysin
Average	26.17391304
St. dev.	15.27970253
	**H** witness
Average	22.98571429
St. dev.	17.41354866
	
**p** value	0.126540558

**Table 31:** 

	freq. **H** cer.	freq. **H** witn.			normal cer.	normal witn.
6,5	0,0145	0,1286	1	9	0,136765	0,175623
18,5	0,3333	0,2714	23	19	0,276188	0,265947
30,5	0,3768	0,3714	26	26	0,301002	0,250478
42,5	0,1159	0,1286	8	9	0,177039	0,146725
54,5	0,1159	0,0571	8	4	0,056196	0,053456
66,5	0,0145	0,0286	1	2	0,009627	0,012113
78,5	0,0145	0,0000	1	0	0,00089	0,001707
90,5	0,0145	0,0000	1	0	4,44E–05	0,00015
102,5	0,0000	0,0000	0	0	1,2E–06	8,16E–06
114,5	0,0000	0,0000	0	0	1,74E–08	2,77E–07
126,5	0,0000	0,0143	0	1	1,36E–10	5,83E–09
	1,0000	1,0000	69	70	0,957752	0,906207

As (also) emphasized by the histogram below, a statistically significant normality distribution hasn't been observed:

**Fig 11:**
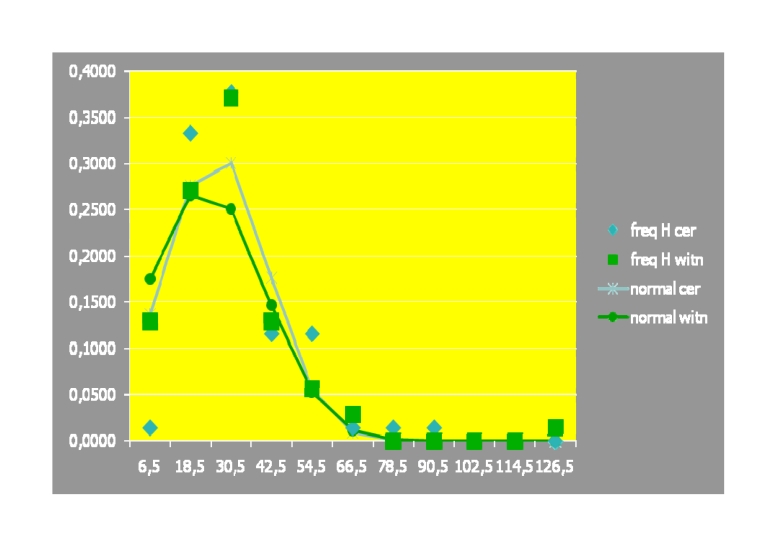
Histogram emphasizing the level of the two studied populations' normality distribution, regarding H

Therefore, we proceeded to CHI2 TEST assay. It resulted p=0,013259, i.e. there has been objectified statistical significant difference, but at limit, between the two lots, regarding H, in ‘favor’ of the Cerebrolysin lot.

**Table 4:** The tabular form of the CHI^2^ TEST results regarding H

**H**	Cerebrolysin	Witness
<11	4	15
11–30	46	39
31–50	14	13
>50	5	3
chi^2^ test, val. **p**	0.013259	
0.5 val. **p**	0.006629	

The interpretation of this discriminative result should be done within a wider, more complex context, i.e. the mean duration of H is, from economical objective reasons, limited, thus having–obviously–including administrative constrains in our clinic activity. Hence, one of the main normal medical criterion to discharge a patient is when he/she reaches a plateau in the actual stage of the rehabilitative process (usually of long term). Therefore, a larger number of H, means the respective patient had a prolonged/ sustained, favorable evolution. 

The two studied populations' normality distribution has also been assessed, regarding the d FIM composed/ exhaustive parameter:

**Table 5:** Normality distribution values of the two assessed populations, regarding d FIM parameter

	freq. **d FIM** cer.	freq. **d FIM** witn.			normal cer.	normal witn.
6,5	0,0580	0,0000	4	0	0,013924	0,001594
18,5	0,0000	0,0000	0	0	0,03237	0,009536
30,5	0,0145	0,0143	1	1	0,063202	0,038648
42,5	0,0725	0,0857	0,0000	5	6	0,103636	0,106099
54,5	0,0435	0,1286	3	9	0,142719	0,197308
66,5	0,2609	0,2571	18	18	0,165064	0,248557
78,5	0,1884	0,3000	13	21	0,160332	0,212108
90,5	0,1159	0,1143	8	8	0,130792	0,122613
102,5	0,1304	0,0571	9	4	0,089606	0,048014
114,5	0,0290	0,0000	2	0	0,051558	0,012736
126,5	0,0725	0,0429	5	3	0,024914	0,002289
	1,0000	1,0000	68	70	0,978117	0,999502

As emphasized by the histogram below, a statistically significant normality of distribution has been observed:

**Fig 12:**
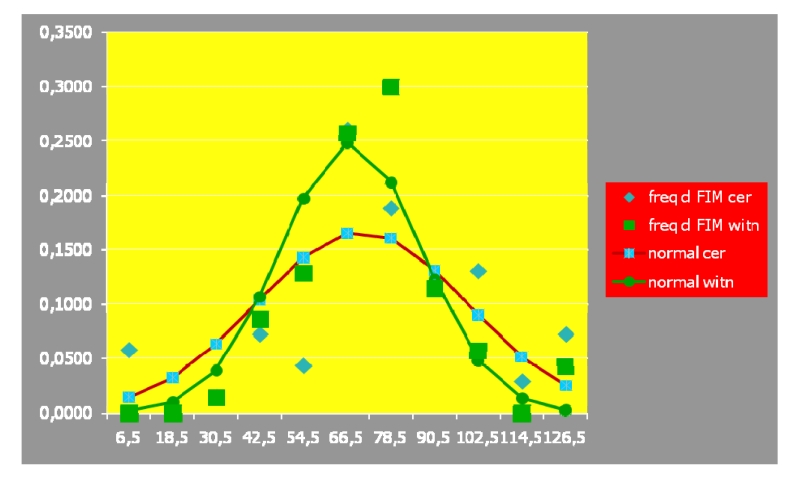
Histogram emphasizing the level of the two studied populations' normality distribution, regarding d FIM

Accordingly, we could use the T TEST; the results below, statistically significant emphasized the lack of difference between (regarding both, the independent and the dependent, related variables) the global/ synthetic outcomes, in the two lots–a FIM (p=0.3682) and d FIM (p=0.2453):

**Table 6:** The tabular form of the T TEST results, regarding a FIM and d FIM

**T TEST**	**a FIM**	**d FIM**
Average	53.36231884	70.5
St. dev.	24.37149433	28.72281
Average	54.52857143	67.61429
St. dev.	15.31005299	19.22807
**p** value	0.368264131	0.245307

Regarding the comparative assessment of the key focused on neuro–motor rehabilitative outcomes, functional/ analytical parameter, KE, related to the frequency distribution, there hasn't been observed a statistically significant populations' normality distribution:

**Table 7:** Normality distribution values of the two assessed populations, regarding KE parameter

	**KE**
Average	10,53623
St. dev.	9,671942
	
Average	11,24286
St. dev.	10,46379
**p** value	0,339934

**Table 71:** 

	freq **KE** cer	freq **KE** witn			normal cer	normal witn
2,5	0,3043	0,4000	21	28	0,087621	0,080677
5,5	0,0435	0,0000	3	0	0,108054	0,098386
8,5	0,0870	0,0429	6	3	0,12103	0,110515
11,5	0,0870	0,0714	6	5	0,123129	0,114343
14,5	0,2174	0,0571	15	4	0,113775	0,108969
17,5	0,0290	0,0429	2	3	0,095488	0,095652
20,5	0,1159	0,1714	8	12	0,07279	0,077337
23,5	0,0435	0,0429	3	3	0,050397	0,057595
26,5	0,0290	0,1143	2	8	0,031693	0,039508
29,5	0,0145	0,0286	1	2	0,018102	0,024962
52,5	0,0290	0,0286	2	2	1,01E–05	4,81E–05
	1,0000	1,0000	69	70	0,822089	0,807992

Consequently, we proceeded to CHI^2^ TEST assay. It resulted a statistically significant difference in favor of the Cerebrolysin treated lot, i.e. the number of days until the first achievement of a functional knee extension in the paretic limb, was significantly shorter in the study lot (p=0,000733):

**Table 8:** The tabular form of the CHI^2^ TEST results, regarding KE

**KE**	Cerebrolysin	Witness
<3	21	28
3–12	18	9
13–22	25	20
>23	5	13
chi^2^ test val. **p**	0,000733	
0.5 val **p**	0,000366	

The same lack of statistically significant populations' normality distribution and consequently, similar mathematical methodology and results, were used/ obtained for another key focused on neuro–motor rehabilitative outcomes, functional/ analytical parameter – WPB (p=0,000000):

**Table 9:** Normality distribution values of the two assessed populations, regarding WPB parameter

	**WPB**
Average	6,942029
St. dev.	9,73152
	
Average	8,871429
St. dev.	11,61823
	
**p** value	0,145084

**Table 91:** 

	freq. **WPB** cerebr.	freq. **WPB** witn.			normal cerebrol.	normal witn.
2,5	0,5652	0,5857	39	41	0,087621	0,080677
5,5	0,0290	0,0000	2	0	0,108054	0,098386
8,5	0,0580	0,0000	4	0	0,12103	0,110515
11,5	0,0580	0,0429	4	3	0,123129	0,114343
14,5	0,0580	0,0286	4	2	0,113775	0,108969
17,5	0,0870	0,0571	6	4	0,095488	0,095652
20,5	0,0725	0,0857	5	6	0,07279	0,077337
23,5	0,0000	0,0286	0	2	0,050397	0,057595
26,5	0,0000	0,1000	0	7	0,031693	0,039508
29,5	0,0000	0,0143	0	1	0,018102	0,024962
52,5	0,0725	0,0571	5	4	1,01E–05	4,81E–05
	1,0000	1,0000	69	70	0,822089	0,807992

**Table 10:** The tabular form of the CHI^2^ TEST results, regarding WPB

**WPB**	Cerebrolysin	Witness
<3	39	39
3–12	11	4
13–22	14	13
>23	5	13
val. of **p**, chi^2^ test	7,75E–06	
0.5 val. of **p**	3,87E–06	

For the other focused on neuro–motor rehabilitative outcomes, functional/analytical parameters, the number of patients suitable to be introduced in histograms, was not enough for concluding results; therefore, we consider the total number of the studied patients within both lots, to be rather small.

Regarding correlation tests in our study, multiple regression analysis mainly evaluated predictor contributivity matters: Hence, the way–of contributivity/ reliability measure–the two analyzed populations aggregate, for (all parameters considered) each studied individual, to the–resulting/ proposed by the EPI INFO soft–optimal appropriate multiple regression formula, regarding the whole predictability level of our study is emphasized in the graphic below:

**Fig 13:**
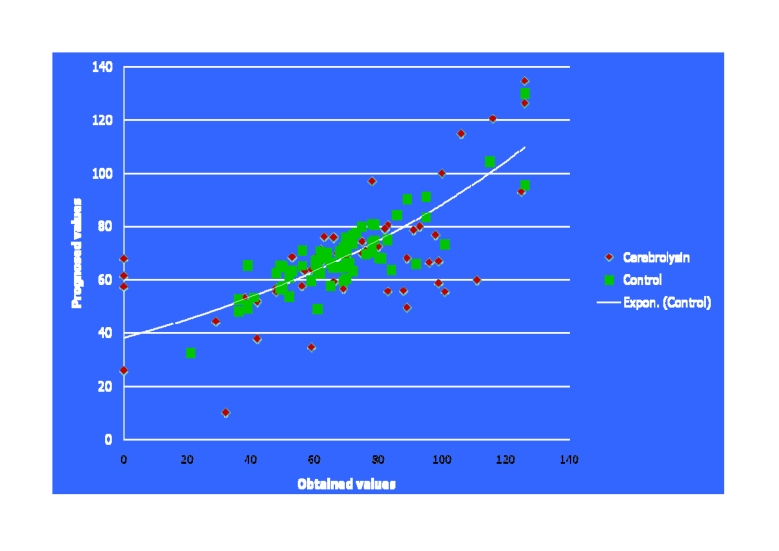
The curve for multiple regression, plotted based on the dependent– independent variables, correlation calculated accounts (mathematical analysis with EPI INFO soft) within our two studied lots

Multiple regression assessment also emphasized the dependent variable d FIM was tightly correlated with the independent variable a FIM (r=0.8660, p=0.0000) in the whole studied population; the significant statistic correlation between d FIM and a FIM (p=0.0000), with its calculated coefficient (r=0.8660), objectifies, at the same time, the importance of the initial correct/ complete clinical and functional evaluation, including its consistent contributivity as a predictor of a complex therapeutic/ rehabilitative (appropriate) management's  efficacy.

Additionally, close to the statistical significance limit, the–focused (but) non functional/ analytical–parameters: PT/KT (r=5.8520, p=0.0115) and H (r=– 4.0070, p=0.0159) were placed, by their predictor contributivity:

**Table 11:** The multiple regression analysis results, regarding the two main– focused (but) non functional/ analytical–parameters: PT/KT and H

	r	p
a FIM	0.8660	0.000000
PT/KT	5.8520	0.011572
H	–4.0070	0.015943
Constant	23.7960	0.005513
Determ. R2	0.5400	

For the other parameters (again) the number of patients in which these could be assessed, was not large enough for statistical contributivity, according to the multiple regression design/ requirements, within these dimensions of the studied lots. 

On the other hand, the statistical significance of the discriminative tests' results that support the hastening, in reaching neurorehabilitative, analytical, outcomes–afore emphasized–effect of Cerebrolysin, represents an–including mathematically based on – strong, objective reason to continue enlarging our studied groups.

## Conclusions

Cerebrolysin administration proved to statistically significant improve the speed of achieving of, at least two key, focused on neuro–motor rehabilitative outcomes, functional/ analytical parameters: KE and WPB; actually, this goes with both, common sense (but mainly based on clinical evidence/expertise) and scientific information: modern pleiotropic drugs–Cerebrolysin is emblematic–obviously cannot cure the CNS lesions (not even provide complete restitution to lost functions)  but they can instead, really hasten recovery. 

Moreover, considering the objectified, imperious necessity to substantially enlarge the studied lots, of our study, we have now initiated a comprehensive, national, 5 year, retrospective, multi–centre (based on unitary data acquisition–see below–frame and mathematical apparatus) study, to evaluate the results of the treatment with Cerebrolysin in traumatic brain injuries (TBI).

**Fig 14:**
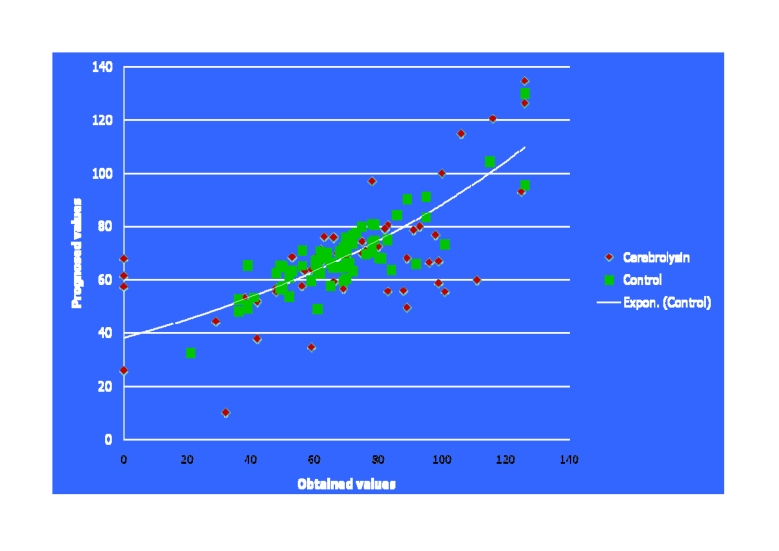
The ‘standardized’, 15 columns table, to summarize, within an unitary data base,  each patient evaluated within the 5 years retrospective, multi-centre, study (by Ebewe coutesy)
